# A Novel Prodrug of a nNOS Inhibitor with Improved Pharmacokinetic Potential

**DOI:** 10.1002/cmdc.202000349

**Published:** 2020-10-21

**Authors:** Cristina Maccallini, Lisa Marinelli, Patrick Indorf, Ivana Cacciatore, Marialuigia Fantacuzzi, Bernd Clement, Antonio Di Stefano, Rosa Amoroso

**Affiliations:** ^1^ Department of Pharmacy University “G. d'Annunzio” of Chieti - Pescara via dei Vestini 31 66100 Chieti Italy; ^2^ Pharmaceutical Institute University of Kiel Gutenbergstraße. 76 24118 Kiel Germany

**Keywords:** acetamidine, amidoxime, mitochondrial amidoxime reducing component, nitric oxide synthase, parallel artificial membrane permeability assay

## Abstract

Under different pathological conditions, aberrant induction of neuronal nitric oxide synthase (nNOS) generates overproduction of NO that can cause irreversible cell damage. The aim of this study was to develop an amidoxime prodrug of a potent nNOS inhibitor, the benzhydryl acetamidine. We synthesized the benzhydryl acetamidoxime, which was evaluated *in vitro* to ascertain the potential NOS inhibitory activity, as well as conducting bioconversion into the parent acetamidine. The prodrug was also profiled for *in vitro* physicochemical properties, by determining the lipophilicity, passive permeation through the human gastrointestinal tract and across the blood‐brain barrier by PAMPA, and chemical, enzymatic, and plasma stability. The obtained data demonstrate that the amidoxime prodrug shows an improved pharmacokinetic profile with respect to the acetamidine nNOS inhibitor, thus suggesting that it could be a promising lead compound to treat all those pathological conditions in which nNOS activity is dysregulated.

## Graphical abstract

**Re‐regulating nNOS**: Amidoxime **2** has been developed as prodrug of a potent nNOS inhibitor, acetamidine **1**. The prodrug was found to be completely converted into the acetamidine after incubation with mARC enzymes. It was profiled for its *in vitro* physicochemical properties, and found to have improved drug‐like properties compared to the parent amidine and promising PAMPA‐BBB penetration.

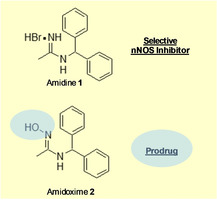

## Introduction

Nitric oxide (NO) is a highly reactive free radical with a short half‐life, that acts as endogenous mediator.^[1]^ Indeed, it freely diffuses through cell membranes, interacting with the soluble guanylyl cyclase (sGS) to regulate blood vessel vasodilatation, as well as with many other cellular targets and pathways, through the generation of a wide pattern of nitrosylated and nitrosated proteins. Basically, low and regulated NO levels ensure a multiplicity of physiological actions in the human body, mainly in the cardiovascular system, in both the central and peripheral nervous systems, and in the immune system. On the other hand, when inappropriate and excessive NO production occurs, NO takes on a pathological role, being associated with the development of different diseases, such as neurological and inflammatory diseases, atherosclerosis, pain and cancer.[^2^, ^3^, ^4^] NO is generated as a product of the enzymatic oxidation of l‐Arg to l‐Cit, by the heme‐containing enzyme nitric oxide synthase (NOS).^[5]^ There are three distinct isoforms of NOS: the endothelial NOS (eNOS) and neuronal NOS (nNOS) are constitutive and calcium dependent„ and catalyze the production of low and pulsating NO levels. Then, there is the calcium independent hinducible NOS (iNOS), which is responsible for the biosynthesis of high and continuous NO levels. eNOS is mainly expressed in endothelial cells, and plays a pivotal role in the control of blood pressure, platelet aggregation, atherosclerosis and angiogenesis.[^6^, ^7^] nNOS is localized in neurons, in epithelial cells of various organs, and in the skeletal muscle. This isozyme has a role in the regulation of neurotransmission, synaptic plasticity, and neural development as well as in the relaxation of nonvascular smooth muscle, and in the cardiovascular function.^[8]^ Unlike the other two isoforms, the expression of iNOS can be induced in different cell types in response to pro‐inflammatory stimuli, such as cytokines, bacterial lipopolysaccharides or other inflammatory agents.^[9]^ Therefore, the NO generated by iNOS is strongly involved in the defense of the organism from pathogens, and in the inflammatory response.[^10^, ^11^]

In mammalian cells, aberrant nNOS induction can have detrimental consequences linked to the overproduction of NO, generating peroxynitrite and nitrosothiols; these highly reactive species can cause irreversible cell damage in excitotoxicity, ischemia, Parkinson's disease, Alzheimer's disease and depression.[^12^, ^13^] Also iNOS induction can cause inflammatory and autoimmune diseases, including septic shock, rheumatoid arthritis, diabetes, multiple sclerosis, and cancer.[^14^, ^15^] The possibility of treating pathological conditions by selective inhibition of nNOS and iNOS could represent a feasible therapeutic strategy, and has stimulated an intense research effort.[^16^, ^17^] On the contrary, NO produced by eNOS has mainly a physiological role, especially in maintaining vascular tone, and its inhibition should be avoided. Several selective nNOS and/or iNOS inhibitors have been published to date, most of them targeting the l‐Arg binding site of the enzyme oxygenase domain, which is highly conserved among the NOS isoforms.[^18^, ^19^, ^20^, ^21^]

**Figure 1:**
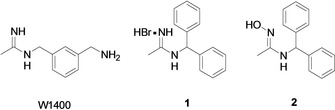
Chemical structures of the iNOS inhibitor **1400 W**, nNOS inhibitor **1** and the designed prodrug **2**.

In our continuous effort to develop selective NOS inhibitors, we have identified several acetamidines structurally related to **1400 W**, a potent inhibitor of the human iNOS (Figure 1),[^22^, ^23^, ^24^, ^25^, ^26^, ^27^, ^28^, ^29^, ^30^] which, however, has never passed clinical trials. In particular, we disclosed the 1‐(benzhydrylamino)ethaniminium bromide (compound **1**) showing high potency toward nNOS and selectivity with respect to the eNOS and iNOS (nNOS IC_50_=0.3 μM; eNOS/nNOS selectivity=1166 folds; iNOS/nNOS selectivity=100 folds).^[31]^ In this molecule the acetamidine moiety is linked to the bulky benzhydryl group, which is responsible for the observed selectivity. Indeed, by means of a docking study, it emerged that compound **1** is unable to fit the iNOS binding cavity, because it is smaller than the nNOS one. On the other hand, it can establish favorable lipophilic interactions within the nNOS, without steric clashes.

Generally, compounds containing an amidino group show high basicity, resulting in low membrane penetration and poor oral absorption. In order to overcome this restriction, the conversion of the amidine moiety into the amidoxime (*N*‐hydroxyamidine) one is a developed strategy to obtain prodrugs of antimalarials, antibacterials, thrombin inhibitors, and antivirals.[^32^, ^33^, ^34^, ^35^] In fact, the amidoxime has an electronegative oxygen atom, which makes it less basic and uncharged at physiological pH with respect to the amidine; consequently, the gastrointestinal (GI) absorption rate and bioavailability are significantly improved. Once absorbed, the amidoxime prodrugs are converted into the corresponding active amidines by means of the mitochondrial amidoxime reducing component (mARC), a molybdenum‐containing enzyme first identified in 2006.[^36^, ^37^] In concert with the electron‐transport proteins cytochrome b5 type B (B5) and NAD(P)H‐dependent cytochrome b5 reductase 3 (B5R), mARC catalyzes the reduction of several N‐oxygenated compounds, like N‐hydroxyguanidines, (sulf‐)hydroxamic acids, hydroxylamines and N‐oxides. There are two coding genes: MARC1 and MARC2, which have a high sequence homology to each other. The endogenous function and physiological role of mARC is still not fully elucidated. All studies performed so far have underlined its role in detoxification of mutagenic and toxic aromatic hydroxylamines and hydroxamic acids.

In order to optimize the pharmacokinetic properties of the nNOS inhibitor **1**, in this study we present the synthesis of amidoxime prodrug **2** (Figure 1), which was *in vitro* evaluated to ascertain the potential NOS inhibitory activity, as well as its bioconversion into the acetamidine **1** by mARC.^[38]^ Compound **2** was also profiled for *in vitro* physicochemical properties, by determining lipophilicity, passive permeation through the human GI tract and blood–brain barrier (BBB) by a parallel artificial membrane permeability assay (PAMPA), and chemical, enzymatic, and plasma stability.

## Results and Discussion

### Chemistry

**Scheme 1:**
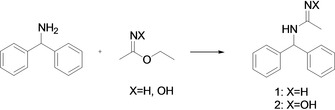
Reaction conditions: solvent free, 90–120 °C; 24–30 h.

The benzyhydryl acetamidine (**1**) and the benzhydryl acetamidoxime (**2**) were synthesized according to Scheme 1. Differently from the previously adopted procedure,^[31]^ compound **1** was obtained from the solvent‐free reaction of benzhydryl amine and ethyl acetimidate hydrobromide, after 24 h under magnetic stirring at 90 °C. Interestingly, the reaction yield was improved with respect to the published synthesis, from 60 to 75 %; also the purification was easier, consisting in a crystallization from water. Similarly, compound **2** was obtained in high purity from the solvent‐free reaction of benzhydryl amine and ethyl acetohydroxamate, after 30 h of magnetic stirring at 120 °C.

### Evaluation of NOS inhibition

The amidoxime **2** was evaluated for its *in vitro* NOS inhibitory activity, by measuring the conversion of l‐Arg to l‐Cit, using a validated HPLC method with fluorescence detection, as previously reported.[^39^, ^40^, ^41^] The human nNOS and iNOS inhibition percent were calculated at 1 μM, while bovine eNOS inhibition was evaluated at 10 μM in order to have an assessment of compound selectivity toward eNOS. Considering the highly conserved primary sequence (>90 %) between the human and bovine eNOS, this latter extremely resembles the human isozyme. In particular, there is only one amino acid variation in a hydrophobic pocket that is on the outside of the heme active site. Therefore the different source of eNOS is usually well accepted and should not affect the inhibitor evaluation.^[42]^ As previously reported, the amidine **1** displayed a good inhibitory potency against nNOS (IC_50_=0.3 μM), and selectivity over iNOS and especially eNOS (1166 folds). Since the NOS inhibition by amidine‐derivatives is strongly related to their basicity and to the interaction with a conserved l‐Glu into the catalytic site, the amidoxime **2**, designed to overcome the high basicity and the permanent positive charge of the amidine **1**, was expected to possess only moderate NOS inhibitory activity. This assumption was verified by the NOS *in vitro* test, which revealed that compound **2** was able to partially inhibit nNOS at 1 μM, giving the 53 % of enzymatic inhibition. This result can be explained considering that the hydroxyl‐group could be able to give the typical hydrogen bonding into the NOS catalytic site, like amidine‐based inhibitors do, resulting in the enzyme blocking. On the contrary, compound **2** was not able to inhibit the eNOS nor the inducible one, in line with the selectivity of the parent compound **1**.

### Bioconversion studies

**Figure 2:**
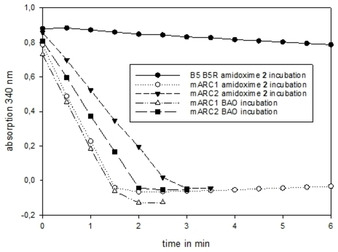
Comparison of the NADH consumption of different incubations of the amidoxime 2 and the model substrate of mARC BAO.

The turnover rate of the amidoxime **2** was tested by an enzymatic NADH assay^[38]^ with the mARC enzyme system in comparison to the model substrate benzamidoxime (BAO). The turnover rate for the amidoxime **2** and BAO are clearly identical for mARC1 and very similar for mARC2. The control experiment with B5 and B5R shows no conversion to the amidine **1** (Figure 2), as expected. These results were confirmed by an LC‐MS experiment.

**Figure 3:**
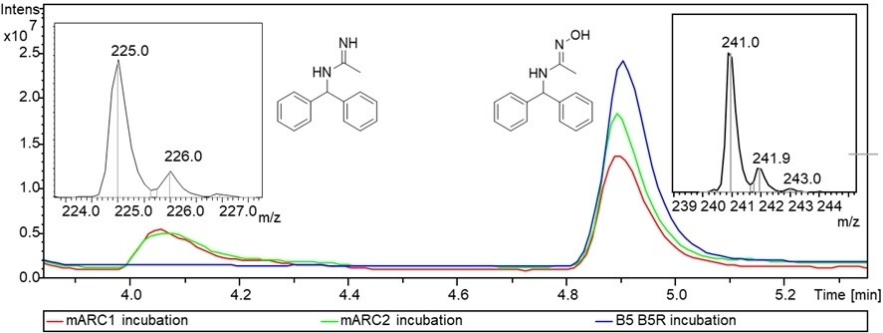
LC‐MS spectrum of incubations with mARC1, mARC2 and B5/B5R. The peak from the reduced amidine **1** appears at 4.05 min and the peak from the amidoxime **2** appears at 4.9 min. Respective mass spectra are shown in the insets.

The reduced amidine **1** could be found in the mARC1 and the mARC2 incubation but not in the control experiment (Figure 3). BAO is an excellent substrate for mARC, which is completely converted to the corresponding benzamidine *in vivo*. Due to this known *in vivo* studies and to other known prodrugs which are activated by mARC, for example, upamostat and ximelagatran,^[38]^ it can be expected that the amidoxime prodrug **2** will also be converted extensively *in vivo*, and is thus a very suitable prodrug for the amidine **1** and making it orally available.

### Evaluation of the physicochemical properties

The herein presented amidoxime **2** was designed as prodrug of the amidine **1** to improve membrane permeability and oral bioavailability. In this context, the deepening of the physicochemical properties is essential to obtain a favorable pharmacological profile. Therefore, we investigated the lipophilicity, the passive permeation through the human GI tract through the PAMPA assays, and the chemical and enzymatic stabilities in simulated GI fluids and plasma. The lipophilicity has proven to be an extremely molecular descriptor in small molecule drug that often correlates well with the biological activity, and the n‐octanol/water partition coefficient (log *P*) can be used as a predictive tool to enable rational design. In this study, the lipophilicity of our compounds was determined by means of the “shake‐flask method” and compared to the corresponding values calculated by the software.^[43]^ However, for ionizable compounds, the distribution coefficient (log *D*) should be used instead of log *P* in the pH range where ionic species exist.^[44]^ In this study, log *P* and log *D* were calculated (Table 1).

**Table 1:** Lipophilicity and PAMPA‐GI and PAMPA BBB permeability assays.^[a]^

Compd	Lipophilicity					PAMPA‐GI permeability Pe [10^−6^ cm s^−1^]^[b]^			PAMPA‐BBB permeability Pe [10^−6^ cm s^−1^]^[c]^
	log *D* 1.4	log *D* 6.5	log *D* 9.4	log *P*	c log *P*	pH 1.4^[d]^	pH 6.5 ^[d]^	pH 7.4^[d]^	
**1**	−0.85 (±0.03)	−0.31 (±0.01)	0.75 (±0.04)	2.54 (±0.62)	2.89	0.57 (±0.03)	1.02 (±0.15)	1.88 (±0.16)	2.7 (±0.09) CNS±
**2**	−0.16 (±0.01)	0.29 (±0.01)	0.39 (±0.02)	3.24 (±0.55)	3.46	0.66 (±0.04)	1.57 (±0.12)	2.03 (±0.16)	5.7 (±2.44) CNS+
**1400 W**	−1.82 (±0.05)	−1.78 (±0.06)	−1.66 (±0.06)	−1.66 (±0.62)	−0.32	0.98 (±0.05)	1.02 (±0.08)	1.86 (±0.75)	1.49 (±0.30) CNS−

[a] Data shown are the means ± SD of three independent experiments;. [b] value <1.5×10^−6^ cm/s, low‐permeability compounds; value >1.5×10^−6^ cm/s, high‐permeability compounds^[46]^; [c] CNS+ (indicative of high BBB permeation): Pe >4.0; CNS± (discrete BBB permeation): Pe from 4.0 to 2.0; CNS− (indicative of low BBB permeation): Pe <2.0; [d] pH of both donor and acceptor compartment.

Results showed that compound **1** is hydrophilic and ionized at all pH values, and showed, as expected, low log *D* values, especially at pH 1.4 and 6.5 (−0.85 and −0.31, respectively); a positive value of log *D* was obtained, albeit very low (0.75), only at pH 9.4. In contrast, the amidoxime 2, that is much less ionized of amidine **1**, displayed higher log *D* values (from −0.16 to 0.39); therefore, the log *P* as free amine was analyzed. Data revealed that amidoxime **2** (log *P*=3.24) was more lipophilic than acetamidine **1** (log *P*=2.54) in accordance with the corresponding calculated log *P* (c log *P*) values (3.46 and 2.89 for compound **2** and **1**, respectively).

PAMPA assay is a high‐throughput technique capable of screening compounds for passive transport across the biological barriers in absence of enzymes and transporters, was selected to predict the membrane permeability of our compounds as it is strongly related to the biological activity. The relative passive transcellular diffusion of compounds, expressed as permeability coefficient (Pe), was determined with a permeability equation.^[45]^ The permeabilities of **1**, **2**, and **1400 W** were determined at pH 5.0, 6.5, and 7.4 to mimic the physiological conditions of the GI tract (Table 1). Data revealed that the obtained Pe values (>1.5×10^−6^) for amidoxime **2** at pH 6.5 and 7.4 were found to be within acceptable range for high‐permeability compounds, suggesting a reasonable predictability with passive absorption at level of the GI tract. On the contrary, compound **1** and **1400 W** displayed good permeability only at pH 7.4. As the new prodrug was designed for the potential treatment of neurodegenerative diseases, an important parameter is its capability to cross the BBB. So, PAMPA‐BBB assay was performed, and results showed that compound **2**, possessing a Pe value higher than 4 compared to the parent compound, may be able to cross the BBB and reach the cerebral area hit by the neurodegeneration process.

The effects of gastrointestinal digestion are important parameters to be taken into consideration in the determination of bioavailability and bioactivity of new compounds. In particular, the luminal instability, due to the pH or enzymatic degradation, may compromise the transmucosal transport of the prodrug designed for permeability enhancement. Therefore, in this study the chemical stability of compound **2** was assessed to exclude any undesirable degradation at acid and physiological environments. Hydrochloric acid buffer (pH 2.0) as a nonenzymatic simulated gastric fluid (SGF), and phosphate buffer (pH 7.4) as nonenzymatic simulated intestinal fluid (SIF) were employed to evaluate the chemical stability. On the other hand, to investigate the ability of amidoxime **2** to be metabolized by enzymes present within the GI tract, its enzymatic stability was checked in vitro at pH 1.3 and 7.4 in the presence of pepsin and pancreatin, respectively.

Finally, the stability in human plasma was assessed. Results showed that amidoxime **2** resulted stable both in acid, physiological, and plasma fluids; in fact, the HPLC chromatograms, obtained after incubation of with the fluids in absence or presence of enzymes, revealed no additional peaks suggesting that the amidoxime **2** was not susceptible to degradation (see Supporting Information).

Based on these results, can be deduced that the investigated compounds possessed good stability in simulated fluids which is predictive of a significant level of protection against a premature chemical and enzymatic degradation in the GI environmental as well as in the plasma. At pH 7.4 they also showed Pe values higher than 1.5×10^−6^ cm/s, revealing a high permeation toward the GI barrier. Moreover, the amidoxime **2**, endowed with the best drug‐like properties (higher lipophilicity and good permeability compared to amidine **1**) could be a suitable compound with the better capability to cross the GI tract after oral administration.

## Conclusion

In conclusion, we have synthesized the benzhydryl acetamidoxime as prodrug of the benzhydryl acetamidine, with the aim to reduce the basicity and improve the absorption rate and bioavailability. The amidoxime was evaluated *in vitro* to ascertain the potential NOS inhibitory activity, revealing only a partial inhibition of nNOS and inactivity toward eNOS and iNOS, and was found completely converted into the corresponding benzamidine after incubation in mARC1 and mARC2. The amidoxime was also profiled for *in vitro* physicochemical properties, showing an amelioration of drug‐like properties (higher lipophilicity, good GI and BBB permeability, and chemical and enzymatic stability) compared to the parent amidine, featuring it as a suitable prodrug orally available. Therefore, based on the obtained results, the benzhydryl amidoxime could be a promising compound to treat all those pathological conditions in which nNOS activity is dysregulated.

## Experimental Section

### Chemistry

All chemicals were purchased from commercial sources and used without further purification. Flash chromatography was performed on silica gel 60 (Merck) and TLC on silica gel 60, F254. Melting points (m.p.) were determined on a Buchi apparatus and given uncorrected. Infrared spectra (IR) were recorded on a FTIR 1600 PerkinElmer spectrometer. NMR spectra were run at 300 MHz on a Varian instrument; chemical shifts (*δ*) are reported in ppm. Elemental analyses were carried out with an Eurovector Euro EA 3000 model analyzer and purity of all compounds was≥95 %. HPLC grade water was obtained by passage through an Elga Purelab water purification system (Elga Labwater,UK). A centrifuge EBA21 (Hettich, Germany) was used.

### Synthesis of 1‐[(diphenylmethyl)amino]ethaniminium chloride (1)

Benzhydrylamine (890 mg, 4.8 mmol) and ethylacetimidate hydrobromide (500 mg, 4.0 mmol) were mixed and allowed to react at 90 °C under magnetic stirring, for 24 h. The obtained pale yellow oil was then dissolved in hot water (10 mL) and the obtained solution was stored at 4 °C, until the desired compound **1** crystallized as a white solid (yield 75 %, m.p. 278–280 °C). ^1^H NMR (DMSO): *δ*=2.26 (s, 3H, CH_3_), 5.60 (s, 1H, CH), 7.29–7.41 (m, 10H, Ar); ^13^C NMR (DMSO): *δ*=19.56, 59.21, 126.16, 128.20, 128.67, 129.01, 129.41, 139.84, 164.45. Anal. calcd. for C_15_H_17_BrN_2_: C 59.03, H 5.61, N 9.18; found: C 58.91, H 5.62, N 9.17.

### Synthesis of (1*E*)‐*N*‐(diphenylmethyl)‐*N′*‐hydroxyethanimidamide (2)

Benzhydrylamine (1600 mg, 8.73 mmol) and ethyl *N*‐hydroxyacetimidate (300 mg, 2.91 mmol) were mixed and stirred at 120 °C for 30 h. The obtained yellow, viscous liquid was purified on silica gel, with CHCl_3_/CH_3_OH (97 : 3) as the mobile phase. Compound **2** was isolated as a yellow solid (yield 54 %, m.p. 112–115 °C). ^1^H NMR (CD_3_OD): *δ*=1,77 (s, 3H, CH_3_); 5,76 (s, 1H, CH); 7,35–7,26 (m, 10H, Ar); ^13^C NMR (CD_3_OD): *δ*=13.77 (CH_3_), 60.19 (CH); 126.84 (Car), 126.90, 127.00, 127.35, 127.60, 128.07, 128.31, 128.62, 142,93, 152,92. Anal. calcd. for C_15_H_16_N_2_O: C 74.97, H 6.71, N 11.66; found: C 75.04, H 6.73, N 11.64.

### Biology

Recombinant human iNOS and nNOS, were purchased from Enzo Life Sciences, Inc. (New York, USA), while bovine recombinant eNOS was purchased from Cayman Chemicals (Ann Arbor, Michigan, USA). HPLC analyses were performed using a Waters system composed of a P600 model pump, a 2475 multi‐fluorescence detector, and a 7725i model sample injector (Rheodyne, Cotati, CA, USA) equipped with a 5 μL loop. The column was a XTerra MS C8 (250×4.6 mm i.d., 5 m particle size; Waters). A column thermostat oven module Igloo‐Cil (Cil Cluzeau Info Labo, France) was used. Chromatograms were recorded on a Fujitsu Siemens Esprimo computer and data were processed by the Empower Pro software (Waters). The appropriate mobile phase was prepared daily, filtered through a 0.45 μm WTP membrane (Whatman, Maidstone, UK), sonicated and degassed before use. Column temperature was kept constant at 20 °C. All assays were performed in triplicate.

### Enzymatic assay for evaluation of NOS inhibition

Enzymes were diluted in 2‐[4‐(2‐hydroxyethyl)piperazin‐1‐yl]ethanesulfonic acid (HEPES) buffer (pH 7.4) to obtain iNOS and nNOS 2.5 μg/mL stock solutions, and eNOS 300 μg/mL stock solution. To measure iNOS activity, 10 μL of iNOS stock solution were added to 80 μL of HEPES buffer at pH 7.4, containing 0.1 mM CaCl_2_, 1 mM d,l‐dithiothreitol (DTT), 0.5 mg/mL bovine serum albumin (BSA), 10 μM flavin mononucleotide (FMN), 10 μM flavin adenine dinucleotide (FAD), 30 μM tetrahydrobiopterin (BH_4_), 10 μg/mL calmodulin (CaM) and 10 μM l‐Arg. To measure eNOS and nNOS activities, 25 μL of enzyme stock solution were added to 75 μL of HEPES buffer pH 7.4, containing 2 mM CaCl_2_, 1 mM DTT, 0.5 mg/mL BSA, 10 μM FMN, 10 μM FAD, 30 μM BH4, 10 μg/mL CaM and 10 μM l‐Arg. Then, 10 μL of a 10 μM (iNOS and nNOS assay) or 100 μM (eNOS assay) solution of the test compound was added to the enzyme assay solution, followed by pre‐incubation of 15 min at 37 °C. The reaction was initiated by the addition of 10 μL of nicotinamide adenine dinucleotide phosphate (NADPH) 7.5 mM (iNOS) or 10 mM (eNOS and nNOS), carried out at 37 °C for 20 min, and stopped by adding 500 μL of ice‐cold CH_3_CN. The mixture was brought to dryness under vacuum and eventually stored at −20 °C, before the fluorescence derivatization. The *o*‐phthalaldehyde‐*N*‐acetylcysteine (OPA/NAC) reagent for fluorescence derivatization was prepared with the molar ratios of 1 : 3, reacting 5 mL of methanolic OPA solution and 20 mL of 0.2 M borate buffer containing 0.1 g of NAC for 90 min to final pH 9.3±0.05. The OPA/NAC solution was stored at 4 °C and saved for no longer than seven days. 600 μL of HPLC grade water was added to the residue of the enzymatic assay and centrifuged at 6000 rpm, Swing‐out Rotor, for 20 min. The fluorescence reaction is realized stirring 190 μL of supernatant and 60 μL of OPA/NAC solution for 5 min.

The HPLC column was eluted at a flow rate of 0.7 mL/min with linear gradients of buffers A (5 % CH_3_CN in 15 mM sodium borate with 0.1 % *v/v* trifluoroacetic acid, pH 9.4) and B (50 % CH_3_CN in 8 mM sodium borate with 0.1 % *v/v* trifluoroacetic acid, pH 9.4). The solvent gradient was 0–20 % B at 0–10 min, B to 25 % at 10–15 min, then to 40 % at 15–20 min and to 70 % at 20–28 min. This composition was maintained until *t*=35 min, before being reduced to the initial 0 % B composition. The injection volume was 5 μL. The fluorescence intensity of the column eluate was monitored at *λ*_ex_=335 nm and *λ*_em_=439 nm.

### Bioconversion studies

#### UV/Vis NADH assay

The assay was performed as previously described.^[38]^ The proteins were dissolved in 20 mM of 2‐(*N*‐morpholino)ethanesulfonic acid (MES) buffer (pH 6.0) in disposable micro UV cuvettes. Each batch contained 15 μg human mARC, 7 μg B5 and 0.16 μg B5R. The enzymes were preincubated with the respective substrate (concentration 3 mM) for 3 min at 37 °C (total volume 240 μL). This mixture was measured as blank value and immediately mixed with 60 μL NADH (concentration 1 mM). The kinetics were measured over a period of 15 min at 37 °C, with absorption between 300 and 400 nm being measured at intervals of 30 sec. The measurement was stopped when the absorption stopped changing. The measurements were taken with a Varian Cary® 50 UV/Vis spectrophotometer from Agilent, which was tempered with a Varian Cary® PCB‐150 Peltier Controlled Cryobath. The samples were mixed with cold methanol and centrifuged for 5 min at 10 000 rpm, Swing‐out Rotor, and stored at −80 °C for further analysis.

#### LC‐MS methodology

A Bruker Amazon SL Ion Trap with upstream Agilent 1260 HPLC system was used with a Waters Xterra MS C8, 4.6×50 mm, 3.5 μm column, with Phenomenex, Security Guard C18 precolumn 3×4 mm. A gradient was employed with eluent A: 0.1 % acetic acid Suprapur, Merck, in Aqua Bidest, eluent B: CH_3_CN, Sigma Aldrich Chromasolv for gradient HPLC. The gradient was changed from 97 % A to 100 % B within 10 min. The injection volume was 1 μL and 10 μL respectively. The column temperature was 20 °C. Software of the HPLC: Hystar 3.2 SR 2. Software to control the mass spectrometer: Bruker Trap Control 7.0 Software to evaluate the data: Bruker Data Analysis 4.0.

#### Chemical‐physical properties determination

Analytical HPLC measurements were run on a Waters 600 HPLC pump equipped with a Waters 2996 photodiode array detector, a 20 μL Rheodyne injector and a computer‐integrating apparatus. The column was a Waters Symmetry RP‐C18 column (4.6×150 mm, 5 μm). The mobile phase was a mixture of TFA 0.01 M/ACN/(MeOH/THF 90 : 10) 20/52/28 and the flow rate was 0.7 mL/min. The UV detector was set at a length of 254 nm.

#### Log *D* and log *P* determination

Both *n*‐octanol and aqueous buffer (pH 1.4, 6.5 and 9.4) solutions were saturated of each other. 2 mg of tested compound was dissolved in 500 μL of organic phase and mixed with an equal volume of aqueous buffer by repeated inversions of up to 200 times for 5 min and then allowed to stand for 30 min for the phases to fully separate. Thereafter, the respective phases were analyzed by HPLC. The c log *P* values were calculated using the ACD LogP software package, version 4.55 (Advanced Chemistry Development Inc., Toronto, Canada).

#### PAMPA‐GI assay

A 2 % solution (w/v) of egg lecithin (phosphatidyl choline >98 %) in dodecane was employed as an artificial membrane solution. Each donor filtration plate well, carefully impregnated with 5 L of this solution, was added of 150 μL of 500 mM test compound buffer solutions (phosphate buffer for pH 7.4 and pH 6.5 and hydrochloric acid buffer for pH 1.4). Subsequently, the drug‐filled donor plate was placed onto the acceptor plate prefilled with the same buffer (300 μL) as the acceptor solution. Once replaced the plate lid, the resulting assembled donor‐acceptor plates were incubated at room temperature for 2 h. The concentration in the acceptor and donor solutions were determined by HPLC. The Pe was determined as previously reported.^[47]^

#### PAMPA‐BBB assay

PAMPA‐BBB assays were performed following the same procedure described above for PAMPA‐GI. Briefly, an artificial membrane consisting in a porcine polar brain lipid extract mixture (purchased from Avantis Polar Lipids, Alabaster, AL, USA) and an incubation time of 18 h were adopted as experimental conditions.

#### Chemical and enzymatic stabilities assays

The stock solutions of compounds **1**, **2**, and **1400 W** were prepared by dissolving 1 mg of compound in 1 mL of EtOH and then diluting 1 : 10 in ultrapure water. For chemical stability, a 0.01 M hydrochloric acid buffer (pH 1.3) and 0.01 M phosphate buffer (pH 7.4) were used. To assess enzymatic stability, the same hydrochloric buffer with and without pepsin (10 or 40 mg/mL) and phosphate buffer with and without pancreatin (10 or 40 mg/mL) were used. The reactions were initiated by adding 50 μL of test compound stock solution to 250 μL of aqueous buffer solution, in screw‐capped vial at 37±0.5 °C, and the mixture was shaken for 2 h at 650 rpm. To stop the enzymatic activity, 250 μL of ice cold CH_3_CN containing 0.5 vol % formic acid was added. The mixture was vortexed and centrifuged at 2 °C and 10 000 rpm, Swing‐out Rotor, for 10 min, and the content of tested compound in the supernatant was analyzed by HPLC. Experiments were run in triplicate and the mean values of the rate constants were calculated.

Human plasma was purchased from 3H Biomedical (Uppsala, Sweden). For the determination of enzymatic stability in human plasma, the reactions were initiated by adding 50 μL of compound stock solution to 450 μL of pre‐heated plasma, and incubated at 37±0.5 °C. At appropriate time intervals, 50 μL of the plasma were taken and deproteinized by the addition of 450 μL of CH_3_CN. After mixing and centrifugation for 10 min at 10 000 rpm, the supernatant (5 μL) was analyzed by HPLC, adopting the same chromatographic conditions described for chemical stability evaluations.^[48]^

## Conflict of interest

The authors declare no conflict of interest.

## Supporting information

As a service to our authors and readers, this journal provides supporting information supplied by the authors. Such materials are peer reviewed and may be re‐organized for online delivery, but are not copy‐edited or typeset. Technical support issues arising from supporting information (other than missing files) should be addressed to the authors.

Supplementary
