# Supplementary material for: A Novel Prodrug of a nNOS Inhibitor with Improved Pharmacokinetic Potential
Source: ChemMedChem. 2020 Oct 21;15(22):2157–63. doi: 10.1002/cmdc.202000349 (PMC7756445; doi:10.1002/cmdc.202000349)
Supplement: Supplementary file 1 — Supplementary [file CMDC-15-2157-s001.pdf]

ChemMedChem

Supporting Information

A Novel Prodrug of a nNOS Inhibitor with Improved Pharmacokinetic Potential

Cristina Maccallini, Lisa Marinelli, Patrick Indorf, Ivana Cacciatore, Marialuigia Fantacuzzi, Bernd Clement,* Antonio Di Stefano, and Rosa Amoroso*

SUPPORTING INFORMATION

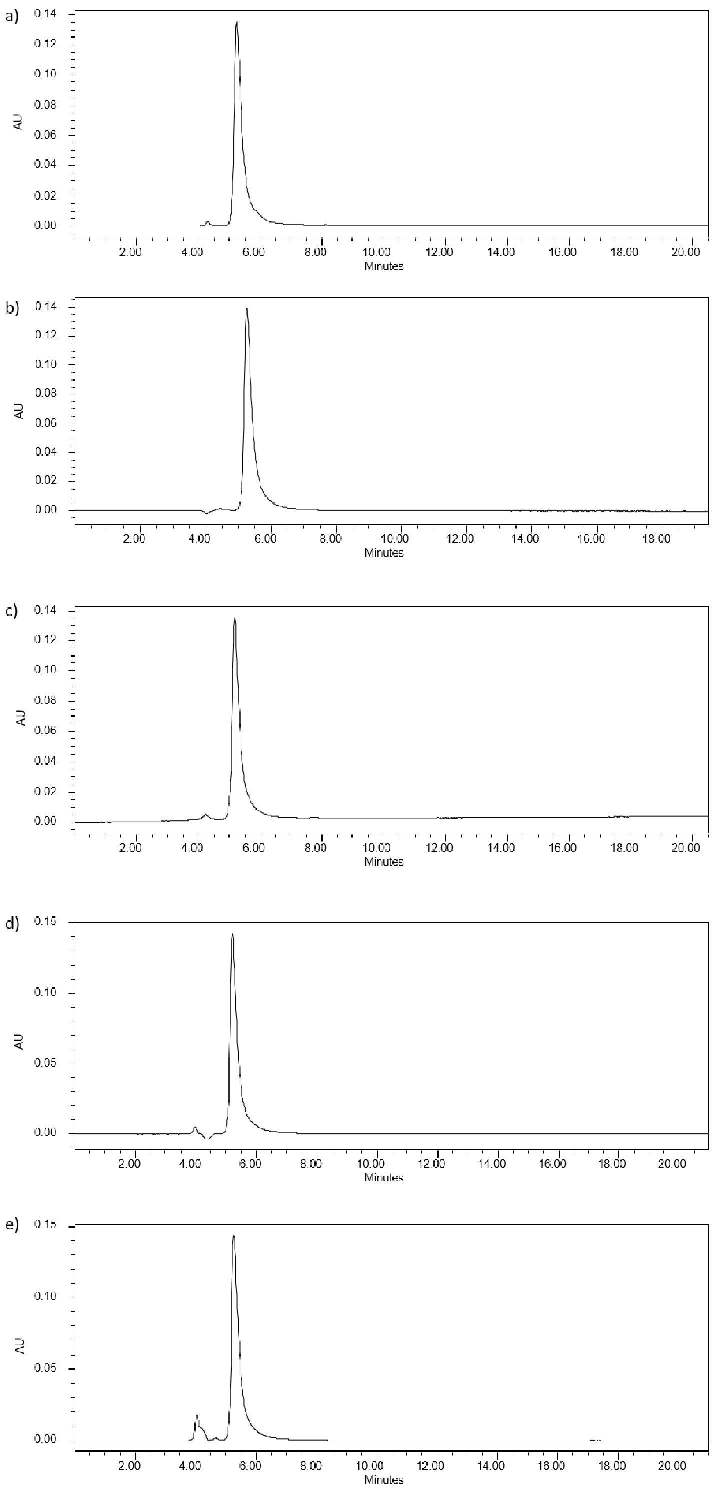

Figure S1. HPLC CHROMATOGRAMS OF AMIDOXIME PRODRUG **2**. Incubation of amidoxime prodrug **2** in: SGF(a) and SIF (b) without enzymes, SGF (c) and SIF (d) in the presence of pepsin and pancreatin (40 mg/mL), respectively, and human plasma (e).
